# Effects of Family Intervention on Physical Activity and Sedentary Behavior in Children Aged 2.5–12 Years: A Meta-Analysis

**DOI:** 10.3389/fped.2021.720830

**Published:** 2021-08-11

**Authors:** Ting Huang, Guanggao Zhao, Haoyuan Tan, Hua Wu, Jinmei Fu, Shunli Sun, Wendi Lv, Zihao He, Qiming Hu, Minghui Quan

**Affiliations:** ^1^School of Physical Education, Nanchang University, Nanchang, China; ^2^Clinical Medical College of Acupuncture Moxibusion and Rehabilitation, Guangzhou University of Chinese Medicine, Guangzhou, China; ^3^Rehabilitation Medicine Center, The Second Affiliated Hospital of Jiaxing University, Jiaxing, China; ^4^Jiangxi Sports Science and Medicine Center, Nanchang, China; ^5^School of Kinesiology, Shanghai University of Sport, Shanghai, China

**Keywords:** physical activity, family intervention, meta-analytic review, parents, children

## Abstract

**Introduction:** To use a quantitative approach to examine the effects of family interventions on physical activity (PA) and sedentary behavior (SB) in children aged 2. 5–12 years.

**Methods:** PubMed, OVID, Web of Science, and others were searched from their inception to May 2020. Intervention studies that examined the effects of family interventions on PA among children aged 2.5–12 years were included in this meta-analysis. Lastly, subgroup analyses were conducted to examine the potential modifying effects of family intervention's characteristics and study quality.

**Results:** Eleven articles met the inclusion criteria for this review. Studies investigated a range of PA outcomes, including moderate-to-vigorous PA (MVPA), total PA (TPA), daily steps, and SB levels. Meta-analysis showed that family intervention had a significant effect on PA [standardized mean difference (SMD) = 0.10; 95% CI = 0.01–0.19], especially for daily steps [weight means difference (WMD) = 1,006; 95% CI = 209–1,803], but not for SB (WMD = −0.38; 95% CI = −7.21–6.46). Subgroup analyses indicated the improvements in PA occurred when children were 6–12 years old, intervention focused on PA only, intervention duration ≤ 10 weeks, and “low risk of bias” study performed.

**Conclusions:** Family intervention may be a promising way to promote children's PA levels, especially for daily steps.

**Trial Registration:** Meta-analysis protocol was registered on PROSPERO: CRD42020193667.

## Introduction

Physical activity (PA) is a key factor in children's physical and mental health development (1–3), playing a crucial role in bone development (4, 5), motor ability development (6), and self-esteem cultivation (7, 8). Previous studies have shown that a low PA level and high sedentary behavior (SB) level lead to poor health (9), increase the risk of obesity (10) and coronary heart disease (11) from childhood to adolescence, and raise the risk of PA deficiency in adulthood (12, 13). However, advances in technology, automated household appliances, and convenient ways of traffic have led to a decline in PA and an increase in SB (14). Eighty percent of the world's children do not meet the PA recommendation from the World Health Organization (15). Therefore, identifying the effective ways to promote children's PA levels has great public health significance.

The family-centered intervention model is designed to interact, purposefully and systematically, with participants and their family members in family settings, to help prevent and respond to various physical and mental health problems (16). Potential mechanisms of intervention effectiveness include the construct of familial or parental social support, the theoretical and practical guidance of PA and SB to families, the technical and logistical support for parents and children activities, and the role modeling and supervision of parents. Family System Theory also believes that the PA and SB behaviors of family members influence each other (17), and parental involvement is crucial in supporting and managing children's related behaviors (PA, SB, diet, screen time, sleep) (18–22). Based on the Family System Theory, some scholars tried to apply family intervention in the field of PA promotion in children (23–25). Some studies found that family interventions can have a significant effect on increasing children's PA and decreasing SB levels (26–28), but in other studies the positive effect was not observed (29–32). Although previous qualitative reviews examined the effects of family intervention on PA and SB levels in children (22, 33), no quantitative review based on experimental studies has been conducted. Therefore, this study aims to identify the effects of family interventions on PA and SB levels in children aged 2–12 years by a using meta-analytic approach. The findings of this study will provide a reference for children's health care work.

## Methods

### Protocol and Registration

This research program has been registered on the PROSPERO System Evaluation Registration Platform, registration number: CRD42020193667. This study has been reported according to the preferred reporting items for systematic reviews and meta-analyses (PRISMA) guidelines (34).

### Data Sources and Search Strategy

Studies were identified by structured database searching from inception until May 2020. Studies were gathered using the following databases: PubMed, OVID, Web of Science, Scopus, and China National Knowledge Internet (CNKI). The following search strings were employed:

(1) Participants: “child^*^,” “preschool,” “kindergar^*^,” “pediatric,” “young child^*^,” “schoolage^*^,” “nursery school^*^,” “primary school^*^,” “grade school^*^,” “elementary school,” “school^*^,” “elementary student^*^,” etc.
(2) Interventions: “intervention,” “health promotion,” “family,” “family-based,” “parent^*^,” “parent-based,” “home-based,” “mother^*^,” “father^*^,” “primary care giver^*^,” “preventi^*^,” “behavio^*^,” “behavior Change^*^,” “treatment,” “methods,” etc.
(3) Outcome: “physical activity,” “exercise^*^,” “sport^*^,” “healthy lifestyle^*^,” “activity^*^,” “inactivity^*^,” “step,” etc.
(4) Study design: search words include “random^*^,” “control^*^,” “trial,” “comparison,” “RCT (randomized controlled trials),” etc.

The exact terms were searched by “OR,” different terms were searched by “AND”. Then the references in the retrieved documents were browsed and conducted a manual retrieval, and supplemented the missing documents in the retrieval process.

### Inclusion and Exclusion Criteria

Inclusion criteria: (1) Participants: children aged 2.5–12 years, basing on PubMed MeSH term definition of preschoolers (2–5 years) and children (6–12 years); (2) Interventions: family Interventions (e.g., intervene in the family, intervene with parents); (3) Outcomes: indicators include PA (including any intensities PA or steps) and SB levels; (4) Study design: randomized controlled trials (RCT) or clinical controlled trials (CCT); (5) published in peer-review journals; and (6) written in English or Chinese.

Exclusion criteria: (1) studies were review article; (2) studies were missing data of PA level as an outcome; (3) participants had physical diseases or dyskinesia; and (4) publications from the same project with a relatively small sample size.

### Data Extraction and Management

Two authors (TH and ZH) and a trained research assistant separately categorized all articles and extracted data. Disagreements were resolved through discussion until there was 100% agreement. The following information was extracted: (1) studies characteristics (e.g., title, authors, publication year); (2) participant's characteristics [e.g., age, body mass index (BMI), sample size]; (3) measuring methods and outcomes; (4) types of interventions; (5) intervention focus; (6) intervention duration; and (7) the mean and standard deviation values of pre- to post-intervention differences between treatment and control groups. If there were multiple results of the same study (e.g., report both any intensities PA and steps), their data were considered as an independent study for data analysis. In the case of missing data, this information was requested from the authors a minimum of three times over four weeks.

### Quality Assessment

Risk assessment was carried out using the Cochrane Risk of Bias tool (35). The evaluation included (1) Random sequence generation, (2) Allocation concealment, (3) Blinding of personnel, (4) Blinding of outcome assessment, (5) Incomplete outcome data, (6) Selective reporting, and (7) Other bias. The evaluation criteria are as follows: the “√” judgment is a low risk of bias, the “×” judgment is a high risk of bias, and the “?” judgment is an unclear risk of bias. Each study was based on an overall assessment of seven items, with a rating of high, moderate, and low risk. Two authors (TH and ZH) and a trained research assistant separately estimate and cross-audit all articles using unified standards. Disagreements were resolved through discussion until there was 100% agreement. Statistical charts of risk bias were generated by RevMan 5.3 software.

### Statistical Analysis

In this review, a random-effect model was used for meta-analysis of the included studies, and STATA was used for analysis. The primary analysis processes included forest map analysis, heterogeneity test, and subgroup analysis. Statistical analysis of data from different units was performed using a 95% confidence interval (95% CI) standardized mean difference (SMD). The values of the effect size were quantified as large (≥0.8 SMD), medium (0.5 SMD– <0.8 SMD), small (0.2 SMD– <0.5 SMD), or non-significant (<0.2 SMD) (36). The weight means difference (WMD) of 95% CI was used for statistical analysis of data of the same unit. *P* < 0.05 was regarded as a significant difference. Depending on the characteristics of included studies, the subgroup analysis was conducted by outcomes, age, BMI, types of intervention, contents of intervention, intervention duration, measuring methods, and study quality to test whether there were differences in the effects among different subgroups.

*I*^2^ statistics were used to test the between-study heterogeneity. When *I*^2^ <25%, 25–<50%, 50– <75% and ≥75% (37), it was defined as very low, low, moderate, and high heterogeneity, respectively. The Egger's test examined publication bias. Sensitivity analysis was conducted to test the robustness of the results, by replacing the fixed-effects model with the random-effects model and removing one study at a time to test whether a single study significantly modified the pooled effect.

## Results

### Study Selection

**Figure 1:**
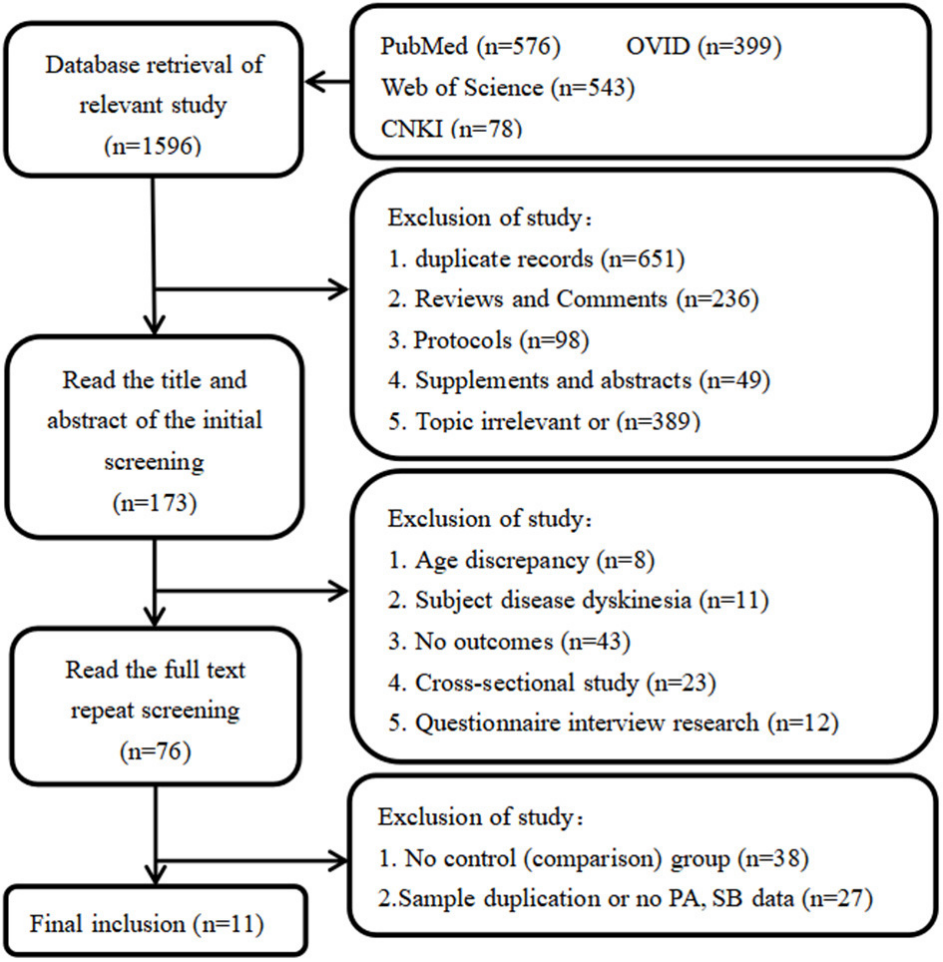
Article selection flow chart for the meta-analysis.

A total of 1,596 articles were searched from each database, 1,585 articles were excluded according to the inclusion and exclusion criteria. Finally, 11 articles were included in this study (31, 38–47) (Figure 1).

### Study Characteristics

All of the included studies were published in 2012 or later, among which seven were published in 2015 or later. Of them, four studies from Australia (38, 40, 45, 46), three from the United States (31, 42, 44). The United Kingdom (47), Germany (43), Finland (41), Norway, and Sweden (39) each have one study. The included studies consisted of 10 RCTs (31, 38–41, 43–47) and 1 CCT (42), with a total of 955 participants in the treatment group and 931 participants in the control group. Five of the included studies (31, 38, 39, 42, 44) only used theory interventions, including PA knowledge education, health behavior lectures, PA counseling services, interviews, and telephone return visits. One of the included studies (41) only used behavior interventions in the specific activity tasks or activity classes that parents and children participated in together. In addition, five of the included studies (40, 43, 45–47) used both theory and behavior interventions. Most interventions included in this review targeted more than one health behavior, and intervention focus was categorized as “PA only” and “included other behavior”. Intervention focus, “PA only,” focuses only on PA improvement during the intervention, not other health behaviors. “Included other behavior” focuses not only on PA but also on improving other health behaviors (e.g., diet, sleep, screen time) (Table 1).

**Table 1:** Characteristics results of a meta-analysis on the family intervention on PA and SB in children aged 2.5–12 years.

**Reference**	**Year of publication**	**Study location**	**Age Mean ± SD**	**BMI**	**Sample**		**Scheme^**C**^**	**Scheme** ^****T****^		**Focus** ^**T**^ ^**3**^		**Intervention duration**	**Outcome**	**Measuring methods^**4**^**
					**^**T**^**	**^**C**^**		**Theory^**1**^**	**Behavior^**2**^**	**PA**	**In+O**			
Tucker et al. (44)	2019	USA	3.6 ± 1.0	Overweight/ Obesity	47	58	Daily PA	√			√	6 mo	MVPA	Questionnaire
Yoong et al. (38)	2019	AUT	4.3 ± 0.5 ^T^ 4.5 ± 0.6 ^C^	Normal	37	37	Daily PA	√			√	3 mo	MVPA, TPA	Accelerometer
Morgan et al. (45)	2019	AUT	4–12	Normal	74	79	Daily PA	√	√	√		2 mo	Daily steps	Pedometer
Laukkanen et al. (39)	2017	NOR and SWE	6.09 ± 1.17 ^T^ 6.5 ± 1.11 ^C^	Normal	44	47	Daily PA	√		√		6 mo	MVPA, SB	Accelerometer
Skouteris et al. (40)	2015	AUT	2.7 ± 0.56 ^T^ 2.8 ± 0.60 ^C^	Normal	71	79	Daily PA	√	√		√	10 we	MVPA, SB	Questionnaire
Tuominen et al. (41)	2015	FIN	6.5 ± 0.5 ^T^ 6.5 ± 0.5 ^C^	Normal	86	89	Daily PA		√	√		7 we	MVPA, SB	Accelerometer
Lloyd et al. (46)	2015	AUT	7.7 ± 2.5	Normal	23	22	Daily PA	√	√		√	7 we	Daily steps	Pedometer
Newton et al. (42)	2014	USA	8.7 ± 1.4	Overweight/ Obesity	13	14	MIG ^5^	√		√		12 we	Daily steps, SB	Pedometer Questionnaire
De Bock et al. (43)	2013	GER	5.0 ± 0.2	Normal	433	376	Daily PA	√	√	√		12 mo	MVPA, SB	Accelerometer
Jago et al. (47)	2013	UK	6–8	Normal	25	23	Daily PA	√	√		√	8 we	MVPA	Accelerometer
Østbye et al. (31)	2012	USA	3.06 ± 1.0	Normal	102	107	Daily PA	√			√	8 mo	MVPA, SB	Accelerometer

*MVPA, Moderate-to-Vigorous physical activity; TPA, total physical activity; SB, sedentary behavior; T, treatment group; C, control group; “mo”; month, “we”, week*.

1 *Theory interventions, including lectures on health behavior education, face to face counsel and various forms PA knowledge education*;

2 *Behavior intervention, including parent-child activity courses or tasks, and intervention measures to complete behavioral tasks*;

3 *Intervention focus were divided into intervention PA only and included other behaviors. e.g. screen time, a healthy diet (increasing the intake of vegetables, fruits, and water, avoiding the intake of junk food, etc.), promoting high-quality sleep, and supporting scientific parenting*.

4 *The measuring method of accelerometer and pedometer is an objective measurement, while the questionnaire is a subjective measurement*.

5 *“MIG” is a minimal intervention group to hand out manuals only. Accelerometers include Actigragh, Kersh Health, Triaxial, and Hookie*.

### Risk of Bias

**Figure 2:**
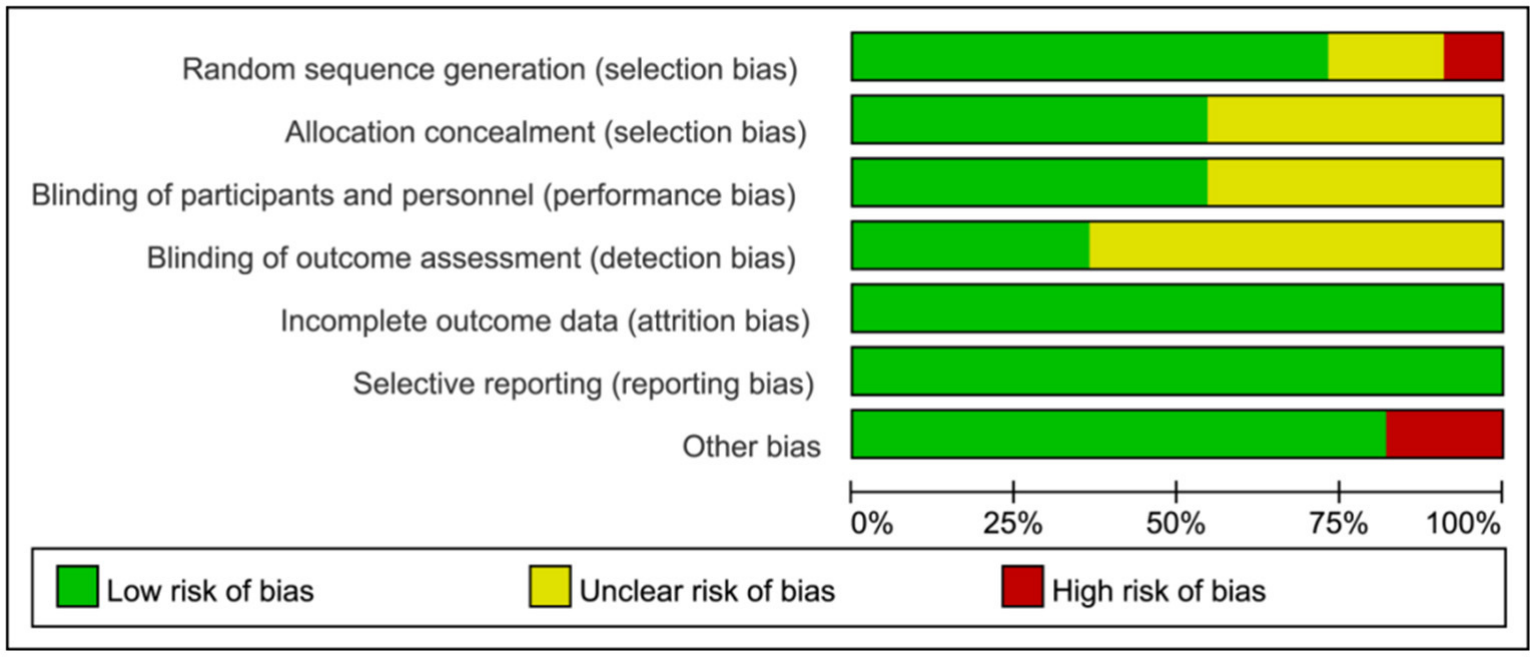
Risk of bias graph each risk of bias item presented as percentages.

**Figure 3:**
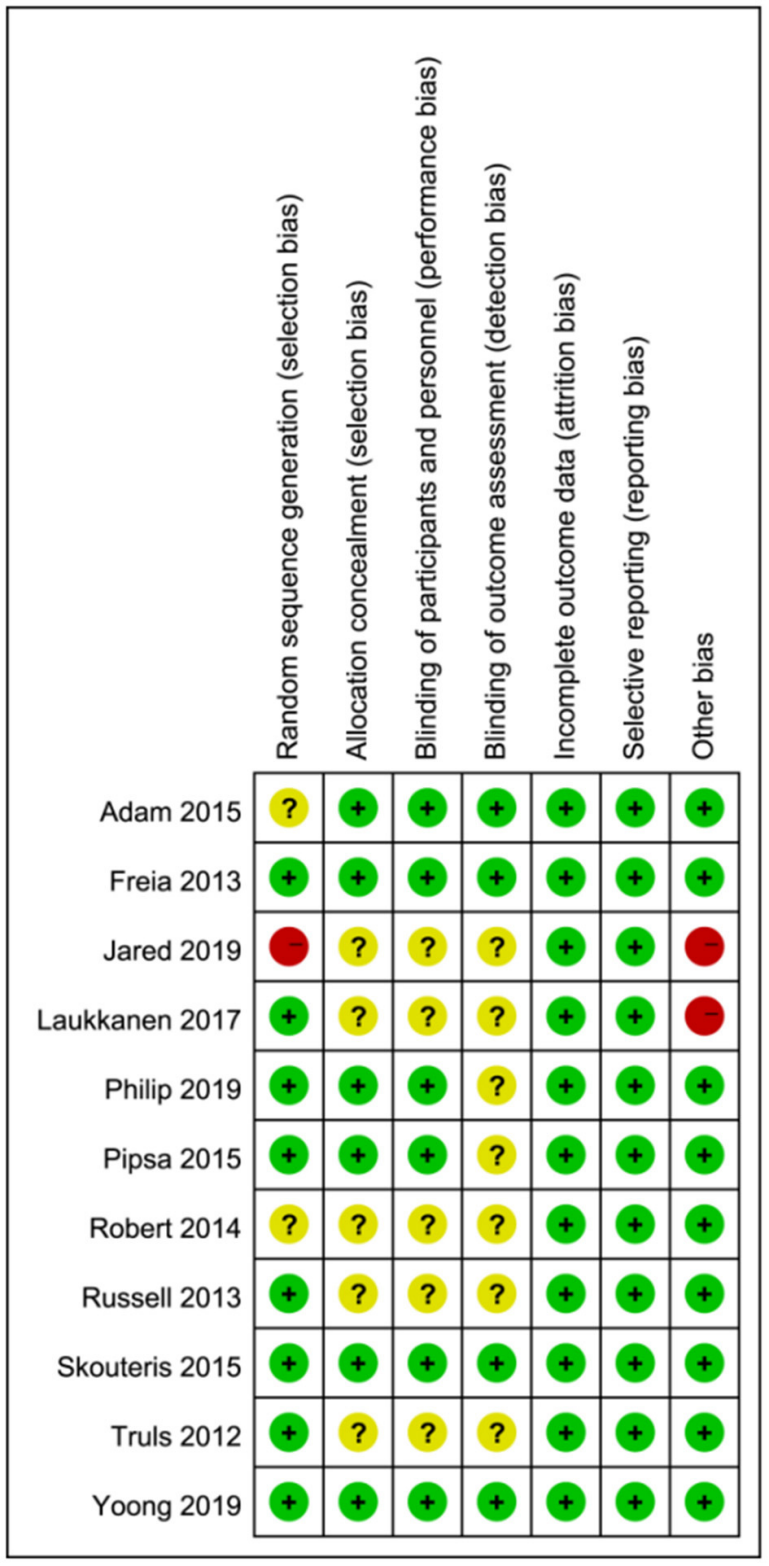
Risk of bias of included studies. (Green = low risk of bias; yellow = unclear risk of bias; red = high risk of bias).

Of the 11 articles, six articles (38, 40, 41, 43, 45, 46) were classified as low risk, two articles (31, 47) were classified as moderate risk, and three articles (39, 42, 44) were classified as high risk. All included studies were non-selective, and the integrity of the data results was described in detail. More than half of all articles described randomization, allocation concealment, and blind implementation (Figures 2, 3).

### Results of Meta-Analysis

#### The Results of PA

**Figure 4:**
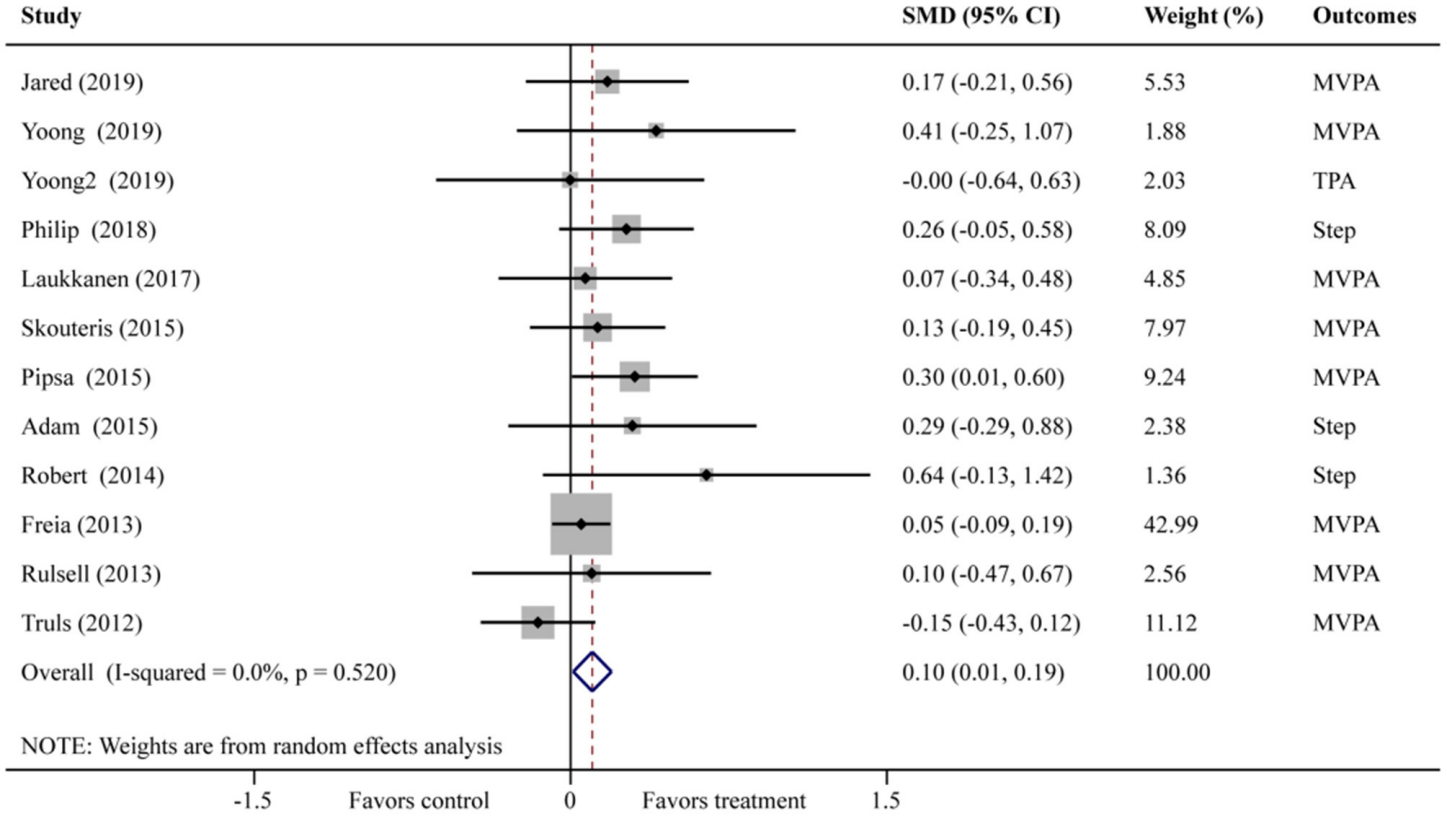
Forest plot of family intervention on PA in children aged 2.5–12 years. (Yoong-MVPA; Yoong2-TPA).

Meta-analysis of 11 included studies was revealed that family intervention had a significant effect on the improvement of PA in children aged 2.5–12 years (SMD = 0.10; 95% CI = 0.01–0.19). Also, no significant heterogeneity was observed across included studies (*I*^2^ = 0%, *P* = 0.52) (Figure 4). Publication bias was also not observed with Egger's test (*P* = 0.11, 95% CI = –0.24–2.08).

Subgroup analysis results showed that subgroups of “daily steps” (WMD = 1,006; 95% CI = 209–1,803), the “≥6 years” (SMD = 0.24; 95% CI = 0.04–0.45), intervention focus “PA only” (SMD = 0.16; 95% CI = 0.01–0.30), intervention duration “ ≤ 10 weeks” (SMD = 0.25; 95% CI = 0.09–0.41), and “low risk of bias” (SMD = 0.13; 95% CI = 0.02–0.23) have significant effect in PA promotion (Table 2).

**Table 2:** Subgroup analysis of the effect of the family intervention on PA.

**Subgroup**	**Potential modifiers**	**No. of studies**	**Effect size (95% CI)**	**Heterogeneity**
All studies		11	0.10 (0.01, 0.19)	*I*^2^*=* 0%, *P =* 0.52
Outcomes^*a*^	MVPA	8	0.43 (−1.19, 2.04)	*I*^2^*=* 19.6%, *P =* 0.27
	TPA	1	–	–
	Daily steps	3	1006 (209, 1803)	*I*^2^*=* 0%, *P =* 0.86
Age^b^	<6 years	5	0.05 (−0.06, 0.15)	*I*^2^*=* 0%, *P =* 0.56
	≥6 years	5	0.24 (0.04, 0.46)	*I*^2^*=* 0%, *P =* 0.71
BMI	Normal	9	0.09 (−0.003, 0.19)	*I*^2^*=* 0%, *P =* 0.53
	Overweight/Obesity	2	0.28 (−0.11, 0.67)	*I*^2^*=* 11.6%, *P =* 0.29
Types of intervention	Theory	5	0.02 (−0.13, 0.27)	*I*^2^*=* 16.4%, *P =* 0.31
	Behavior	1	–	–
	Theory plus behavior	5	0.10 (−0.02, 0.21)	*I*^2^*=* 0%, *P =* 0.74
Intervention focus	PA only	5	0.16 (0.01, 0.30)	*I*^2^*=* 20.6%, *P =* 0.28
	PA plus others	6	0.06 (−0.10, 0.22)	*I*^2^*=* 0%, *P =* 0.59
Intervention duration	>10 we	7	0.08 (−0.04, 0.19)	*I*^2^*=* 7.5%, *P =* 0.37
	≤ 10 we	4	0.22 (0.02, 0.41)	*I*^2^*=* 0%, *P =* 0.84
Measuring methods	Subjective	2	0.15 (−0.10, 0.39)	*I*^2^*=* 0%, *P =* 0.86
	Objective	9	0.11 (−0.004, 0.22)	*I*^2^*=* 9.5%, *P =* 0.36
Risk of bias	Low risk	6	0.13 (0.02, 0.23)	*I*^2^*=* 0%, *P =* 0.62
	Moderate risk	2	−0.11 (−0.35, 0.14)	*I*^2^*=* 0%, *P =* 0.43
	High risk	3	0.19 (−0.08, 0.45)	*I*^2^*=* 0%, *P =* 0.44

a *The subgroup of outcomes units were the same, and WMD statistics were used, SMD was used for all the other subgroup except the outcomes subgroup. Yoong et al. (38) contained two outcomes [MVPA and light PA(LPA)], so the total number of outcomes subgroups was 12*;

b *philip (40) is not divided into age subgroup because of participants were 4-12 years old*.

#### The Results of SB

**Figure 5:**
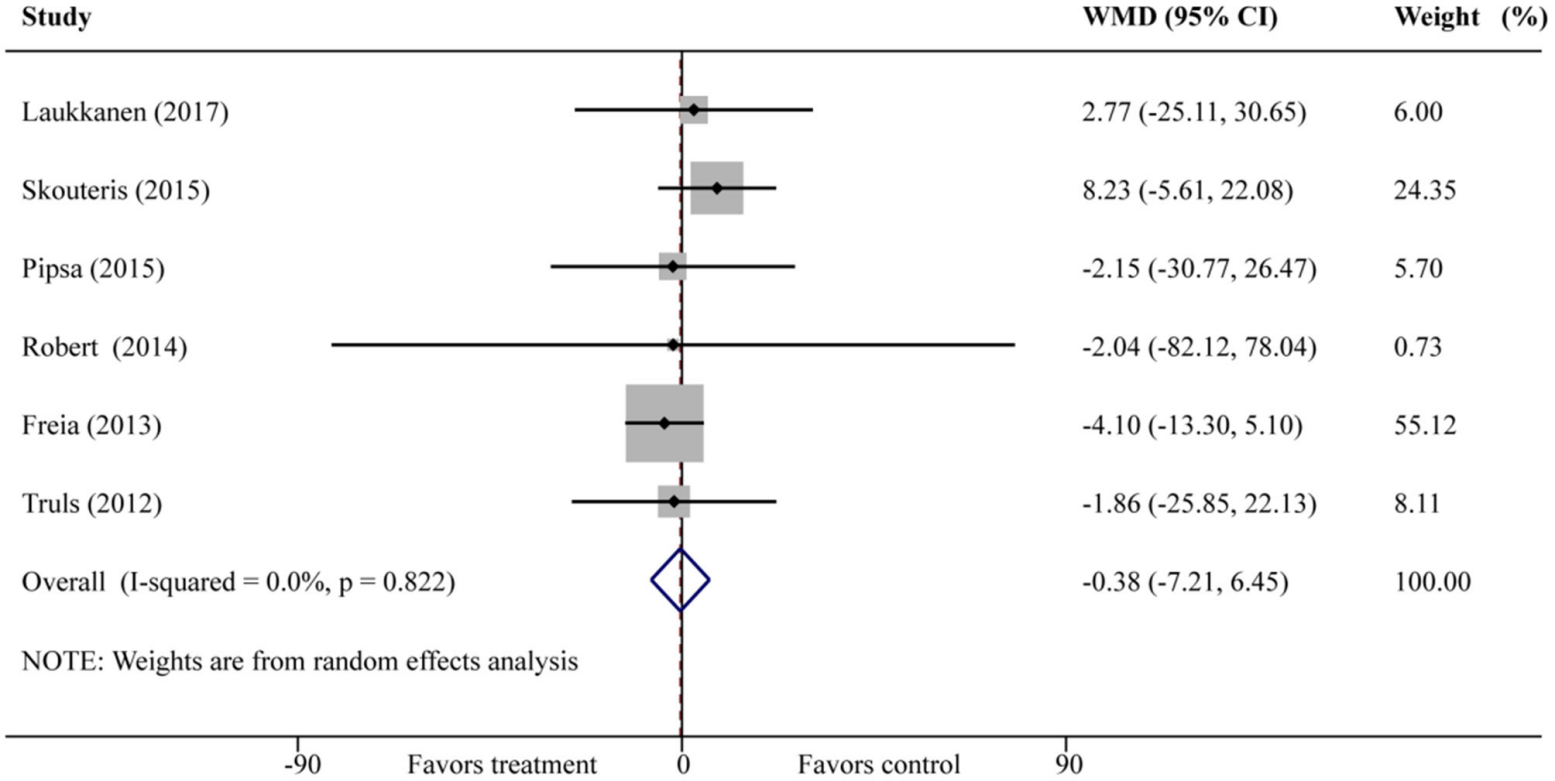
Forest plot of family intervention on SB in children aged 2.5–12 years.

Meta-analysis of 6 included studies was revealed that family intervention had no significant effect on the improvement of SB outcome in children aged 2.5–12 years (WMD =−0.38; 95% CI = −7.21–6.46) (Figure 5). There was no significant difference in all subgroups. Also noteworthy was that no significant heterogeneity was observed (*I*^2^ = 0%, *P* = 0.82) (Table 3). Publication bias was also not observed with Egger's test (*P* = 0.72, 95% CI = −1.36–1.80).

**Table 3:** Subgroup analysis of the effect of the family intervention on SB.

**Subgroup**	**Potential modifiers**	**No. of studies**	**WMD (min/day) (95% CI)**	**Heterogeneity**
All studies		6	−0.38 (−7.21, 6.46)	*I*^2^*=* 0%, *P =* 0.82
Age	<6 years	3	−0.46 (−7.76, 6.84)	*I*^2^*=* 6%, *P =* 0.35
	≥6 years	3	0.23 (−19.15, 19.61)	*I*^2^*=* 0%, *P =* 0.97
BMI	Normal	5	−0.37 (−7.22, 6.49)	I^2^ *=* 0%, *P =* 0.70
	Overweight/Obesity	1	–	–
Types of intervention	Theory	3	−0.004 (−17.73, 17.74)	*I*^2^*=* 0%, *P =* 0.97
	Behavior	1	–	–
	Theory plus behavior	2	−0.32 (−7.99, 7.34)	*I*^2^*=* 52.7%, *P =* 0.15
Intervention focus	PA only	4	−3.30 (−11.62, 5.01)	*I*^2^*=* 0%, *P =* 0.98
	PA plus others	2	5.71 (−6.28, 17.70)	*I*^2^*=* 0%, *P =* 0.48
Intervention duration	>10 weeks	4	−3.23 (−11.40, 4.94)	*I*^2^*=* 0%, *P =* 0.97
	≤ 10 weeks	2	6.27 (−6.20, 18.73)	*I*^2^*=* 0%, *P =* 0.52
Measuring methods	Subjective	2	7.94 (−5.71, 21.58)	*I*^2^*=* 0%, *P =* 0.80
	Objective	4	−3.16 (−11.05, 4.73)	*I*^2^*=* 0%, *P =* 0.97
Risk of bias	Low risk	3	−0.44 (−7.85, 6.96)	*I*^2^*=* 6.1%, *P =* 0.35
	Moderate risk	1	–	–
	High risk	2	2.25 (−24.08, 28.58)	*I*^2^*=* 0%, *P =* 0.91

### Sensitivity Analysis

Two sensitivity analyses were performed to test the robustness of our results: (1) the findings were consistent when the fixed-effects model was replaced by the random-effects model and (2) the results indicated no single study to be significantly modified by the overall trend by removing one study from the meta-analysis each time.

## Discussion

### Overall Effect of Family Intervention

This study aimed to quantitatively examine the effect of family interventions on the PA and SB in children aged 2.5–12 years by synthesizing the available literature in this field of inquiry. Through the combined 11 studies included, we found that family intervention could effectively improve the PA of children aged 2.5–12 years, especially for daily steps, but there was no significant effect on SB.

### Comparison With Previous Findings

Findings of this study indicated that family interventions have a positive effect on PA in children aged 2.5–12 years, and this study is, therefore, a valuable extension of two published systematic reviews and meta-analysis (48, 49). A meta-analysis provides evidence that school-based interventions can be effective in increasing PA enjoyment in children (48). Jane's (49) meta-analysis, based on school and family interventions, found that family interventions (involving children and parents) had better PA improvement than school interventions (only children). On this basis, when this study concentrates on family interventions, it still found that a significant intervention effect on PA in children. This study may provide additional information and contribute to this area of inquiry from family intervention and PA.

Indeed, a growing body of evidence has shown the benefits of intervention on children's PA (47, 48), however, which index of PA is more sensitive to family intervention remains unclear. Among children, previous reviews suggested that neither active play interventions (50) nor school-based interventions (51) affect moderate-to-vigorous PA (MVPA). In accord with previous studies, findings from this study align with the earlier points indicating that family interventions have no effects on MVPA. However, family interventions significantly improved the children's daily steps by 1,006 steps per day. Among previous reviews suggested that positive relationships between daily steps and physical fitness were observed (52). Daily steps are an excellent indicator of health-related outcomes (53, 54). Some studies suggested converting MVPA to steps because daily steps were generally easier to recognize (55). Findings from this study show that have no significant improvement in MVPA but improve daily steps may be the increased activity comes from LPA, not MVPA. Although PA guideline-recommended to engage in sufficient MVPA to obtain health benefits from PA (56), previous reviews revealed that engaging in more LPA is also suitable for children's health (57, 58). Therefore, it cannot be ignored the potential health effects from family interventions to enhance LPA.

Nevertheless, the results of this review showed that family intervention had no significant effect on SB in children aged 2.5–12 years. It is a disappointing outcome for public health practitioners and researchers who consider the family a promising intervention setting (17). Previous school-based (51) or classroom-based (59) interventions have also been ineffective for SB. In general, family interventions design may focus more on PA logically not SB. Future research should consider the differences and concerns between PA and SB in study design.

### Analysis of Influencing Factors

The result of subgroup analysis expressed that family interventions were more effective in increasing PA levels in certain subgroups, for example, intervention focus “PA only,” “low risk of bias”. In addition, this review showed that age might be one of the factors influencing the effectiveness of family interventions. How do these findings compare to those of other published studies? A number of studies focused on preschool children found no changes in PA and SB following PA interventions (60, 61). However, in this study, the family intervention had a significant effect on PA in children aged 6–12 years. With the growth and cognitive development, the cognitive ability of school-age children (6–12 years old) was better than preschool children (62), and they also had a better understanding of the family intervention and PA. At this time, parents could set a good example, or they live in a PA positive family, which can have a profound effect on a child's PA. Therefore, well-designed and targeted RCTs were needed for children of other ages in the future.

The study also demonstrated that intervention duration affects the effectiveness of family interventions. Intervention duration was categorized as “>10 weeks” and “ ≤ 10 weeks” based on characteristics of included studies. It was found that interventions <10 weeks may have a more significant impact on PA improvement. The short-term (≤10 weeks) intervention effects may be attributed to the curiosity of the participants in the early stages of the intervention, and they are willing to participate in the implementation. Over time, the decline in the interest and compliance of the participants led to the intervention effect not being maintained.

### Strength and Limitations

This study has demonstrated several strengths. First, this is the first meta-analysis to quantitatively examine the effect of family interventions on PA in children aged 2.5–12 years, which provides additional insight in the field of family interventions and PA. Second, the meta-analysis is based on data from controlled trials studies regarded as a study design that substantially reduces selection bias and has good comparability.

There were also some limitations in this study. First, most of the included studies were distributed in developed countries, so the research results were not widely representative. However, this study has included as much as possible the latest and most comprehensive research related to this proposition. Second, the family intervention programs (focus, means, duration) varied across included studies, leading to estimation bias of the overall effect. However, sensitivity analysis showed that the reduction of any one of the included studies did not significantly affect the combined results of this study.

## Conclusions

In summary, findings from this meta-analysis that family intervention can effectively improve PA of children aged 2.5–12 years, especially daily steps, but has no noticeable effect on SB. Considering that family members engage in physical activity together is safe, meaningful, and effective for not only promoting the relationship between parents and children but also the development of good habits, we should encourage family members to take up physical exercise together. Future studies should focus on considering the different characteristics of preschoolers and school-age children, exploring the optimal combination of interventions focus, means, and duration.

## Data Availability Statement

The original contributions presented in the study are included in the article/Supplementary Materials, further inquiries can be directed to the corresponding authors.

## Author Contributions

TH, HW, MQ, HT, and ZH designed conception and search strategy. TH, HT, MQ, and ZH designed inclusion and exclusion criteria. TH and ZH conducted the quality assessment with arbitration by TH. Summary statistics were produced by TH and data analysis was performed by MQ and TH. TH, WL, and SS wrote the first draft. All authors made substantive contributions and approved the final manuscript.

## Conflict of Interest

The authors declare that the research was conducted in the absence of any commercial or financial relationships that could be construed as a potential conflict of interest.

## Publisher's Note

All claims expressed in this article are solely those of the authors and do not necessarily represent those of their affiliated organizations, or those of the publisher, the editors and the reviewers. Any product that may be evaluated in this article, or claim that may be made by its manufacturer, is not guaranteed or endorsed by the publisher.
