# Supplementary figures and images for: Effects of Family Intervention on Physical Activity and Sedentary Behavior in Children Aged 2.5–12 Years: A Meta-Analysis
Source: Front Pediatr. 2021 Aug 11;9:720830. doi: 10.3389/fped.2021.720830 (PMC8384957; doi:10.3389/fped.2021.720830)

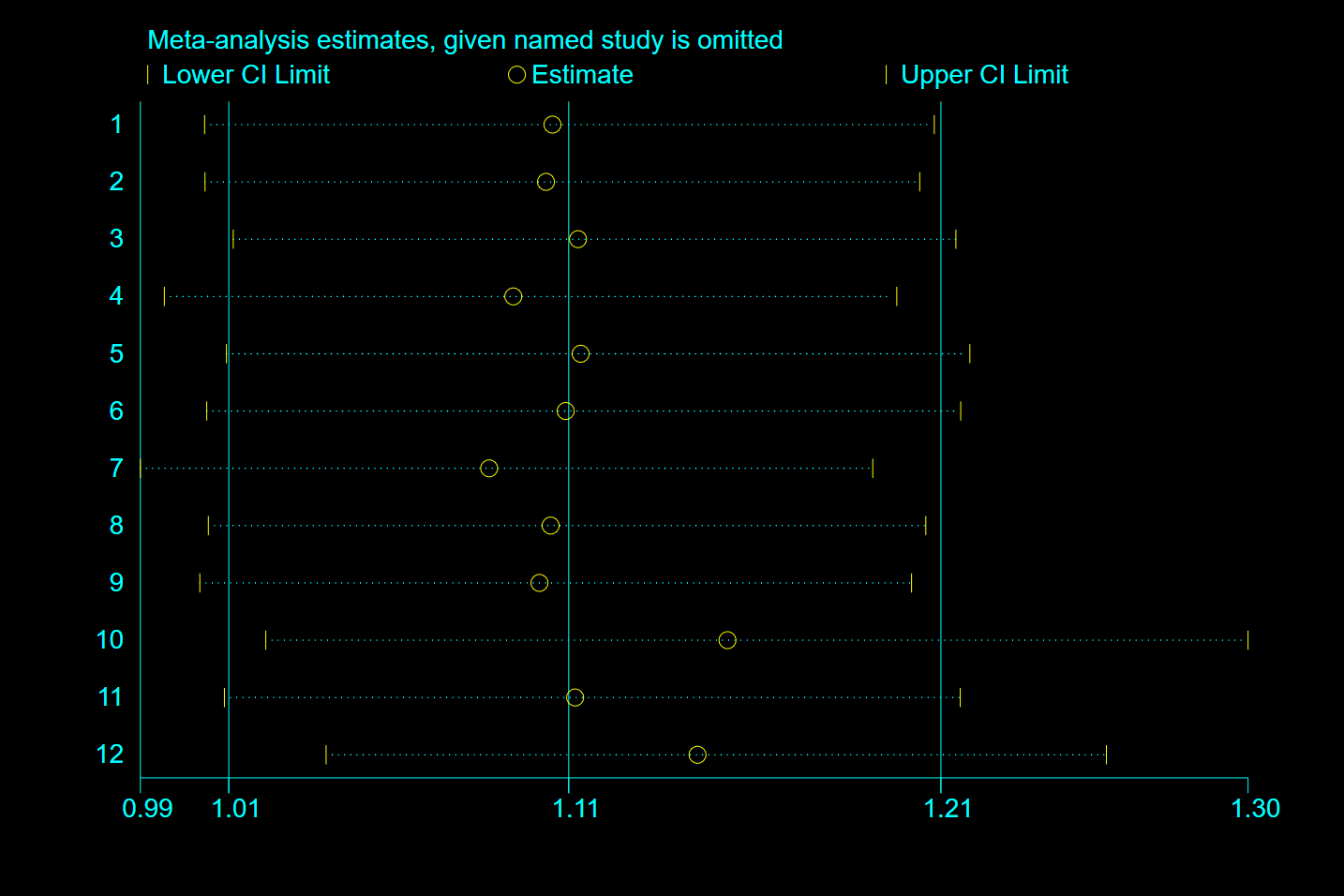

Supplement: Supplementary file 1 [file Image_1.PNG]

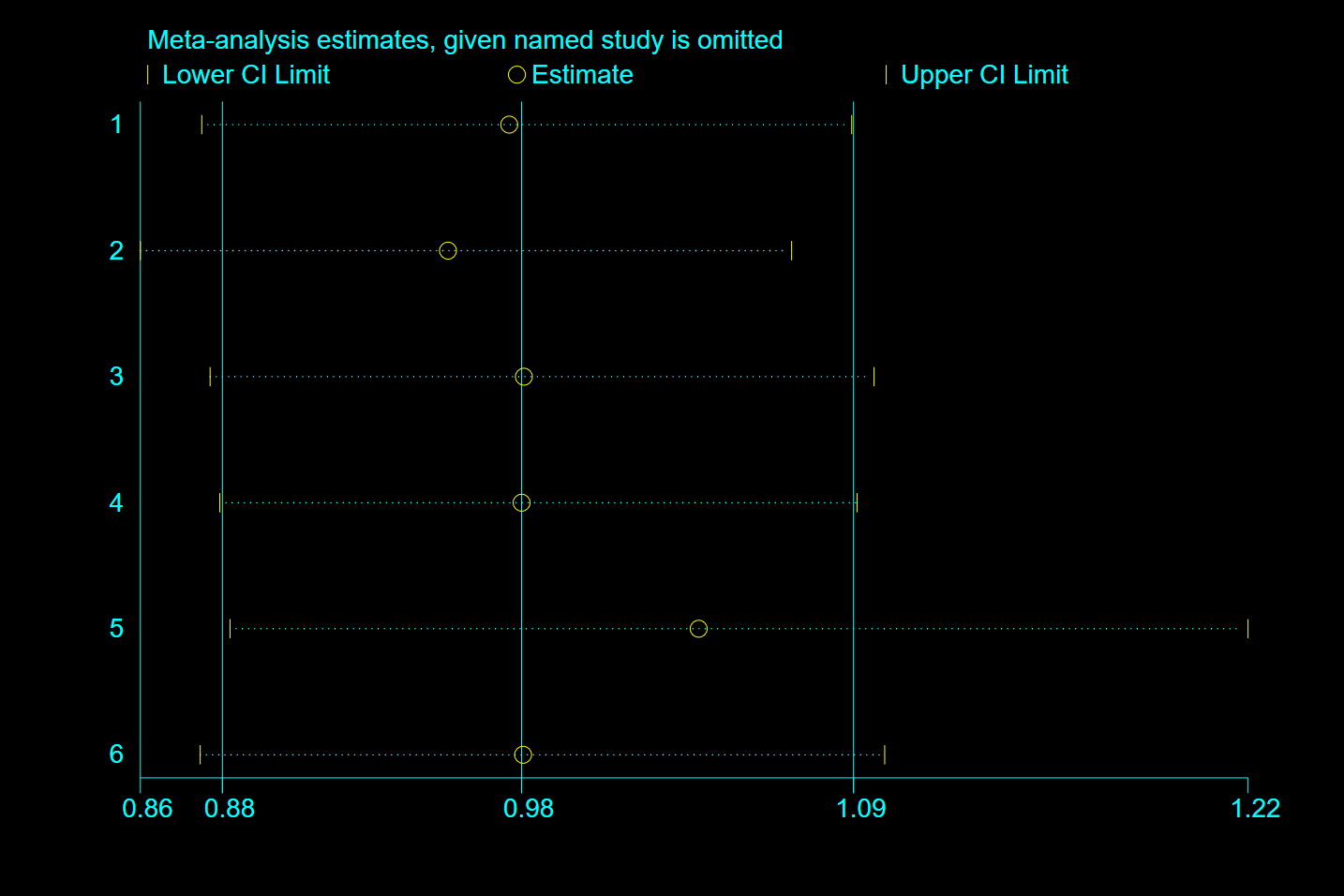

Supplement: Supplementary file 2 [file Image_2.PNG]
