# Supplementary material for: Effects of Family Intervention on Physical Activity and Sedentary Behavior in Children Aged 2.5–12 Years: A Meta-Analysis
Source: Front Pediatr. 2021 Aug 11;9:720830. doi: 10.3389/fped.2021.720830 (PMC8384957; doi:10.3389/fped.2021.720830)
Supplement: Supplementary file 3 [file Table_1.DOC]

| **Section/topic** | **#** | **Checklist item** | **Reported on page #** |
| --- | --- | --- | --- |
| **TITLE** | | |  |
| Title | 1 | Effects of Family Intervention on Physical Activity and Sedentary Behavior in Children Aged 2-12: A Meta-Analysis | 1 |
| **ABSTRACT** | | |  |
| Structured summary | 2 | Objective: To use a quantitative approach to examine the effects of family interventions on physical activity (PA) and sedentary behavior (SB) in children aged 2-12 years.  Methods: PubMed, OVID, Web of Science, and others were searched from their inception to May 2020. Intervention studies that examined the effects of family interventions on PA among children aged 2-12 years were included in this meta-analysis. Lastly, subgroup analyses were conducted to examine the potential modifying effects of family intervention’s characteristics and study quality.  Results: Eleven articles met the inclusion criteria for this review. Studies investigated a range of PA outcomes, including moderate-to-vigorous PA (MVPA), total PA (TPA), daily steps, and SB levels. Meta-analysis showed that family intervention had a significant effect on PA [standardized mean difference(SMD)=0.10; 95%CI=0.01-0.19], especially for daily steps [weight means difference(WMD)=1,006; 95%CI=209-1,803], but not for SB (WMD=-0.38; 95%CI=-7.21-6.46). Subgroup analyses indicated the improvements in PA occurred when children were 6-12 years old, intervention focused on PA only, intervention duration ≤ 10 weeks, and “low risk of bias” study performed. Conclusions: Family intervention may be a promising way to promote children’s PA levels, especially for daily steps. | 2 |
| **INTRODUCTION** | | |  |
| Rationale | 3 | It is encouraging to note that experts and scholars are attaching increasing importance to research on the improvement of PA through family intervention, but the results are not the same. Some studies have found that family intervention has a significant effect on increasing children's PA and decreasing SB levels, but in some studies, the result is the family intervention has no effect on improving children's PA and SB. In recent years, although qualitative reviews examined the effects of family intervention on on PA and SB levels in children, no quantitative review based on experimental studies has been conducted. Therefore, this study aims to identify the effect of family intervention on PA and SB levels in children aged 2-12 years, by using meta-analytic approach. Findings of this study will provide a reference for children's health care work. | 3 |
| Objectives | 4 | 1. Participants: children aged 2-12 years, basing on PubMed MeSH term definition of preschoolers (2-5 years) and children (6-12 years); 2) Interventions: family Interventions (e.g., intervene in the family, intervene with parents); 3) Outcomes: indicators include PA (including any intensities PA or steps) and SB levels; 4) Study design: randomized controlled trials (RCT) or clinical controlled trials (CCT); 5) published in peer-review journals; 6) written in English or Chinese. | 4 |
| **METHODS** | | |  |
| Protocol and registration | 5 | These records have been registration. CRD42020193667  Web: https://www.crd.york.ac.uk/prospero/#searchadvanced |  |
| Eligibility criteria | 6 | Inclusion criteria: 1) Participants: children aged 2-12 years, basing on PubMed MeSH term definition of preschoolers (2-5 years) and children (6-12 years); 2) Interventions: family Interventions (e.g., intervene in the family, intervene with parents); 3) Comparisons: the control group as the daily PA or other intervention; 4) Outcomes: indicators include PA (including any intensities PA or steps) and SB levels; 5) Study design: RCT or controlled study; 6) published in peer-review journals.  Exclusion criteria: 1) studies were not written in English or Chinese; 2) studies were review article or studies were missing PA or SB changed data; 3) participants had physical diseases or dyskinesia; 4) publications from the same project with relatively small sample size; 5) Studies were significant differences in PA or SB measurements at baseline. | 5-6 |
| Information sources | 7 | PubMed, OVID, Web of Science, Scopus, and the China National Knowledge Infrastructure were searched from their inception to May 2020. | 4 |
| Search | 8 | The following search strings were employed:  1) Participants: preschoolers, school-age children. search term include "child*", "preschool", "kindergar*", "pediatric", "young child*", "schoolage*", "nursery school*", "primary school*", "grade school*", "elementary school", "school*", "elementary student*", etc.  2) Interventions: Family intervention, Search words include "intervention", "health promotion", "family", "family-based", "parent*", "parent-based", "home-based", "mother*", "father*", "primary care giver*", "preventi*", "behavio*", "behavior Change*", "treatment", "methods", etc.  3) Outcome: physical activity. English search words include "physical activity", "exercise*", "sport*", "healthy lifestyle*", "activit*", "inactivit*", "step", etc.  4) Study design: randomized controlled trial or controlled study. Search words include "random*", "control*", "trial", "comparison", "RCT", etc. Then I browse the references in the retrieved documents and conduct manual retrieval, and supplement the missing documents in the retrieval process. | 4 |
| Study selection | 9 | A total of 1596 articles were searched from each database, The literature was selected according to inclusion and exclusion criteria. Read the title and abstract of the initial screening. Read the full text repeat screening. | 7 |
| Data collection process | 10 | The data were extracted separately by two researchers. If they have a dispute, the question will be passed to a third researcher and the result will be decided in a group discussion. If there were multiple results of the same study (e.g., report both MVPA and steps), their data were considered as independent study for data analysis.If the data was missed, contact the author for access. | 5-6 |
| Data items | 11 | We extracted the following information: (1) studies characteristics (e.g., title, authors, publication year); (2) participants characteristics (e.g., age, BMI, sample size); (3) measuring methods and outcomes; (4) means of intervention; (5) contents of intervention; (6) intervention duration; and (7) the mean and standard deviation values of pre- to post-intervention differences between treatment and control groups. | 5-6 |
| Risk of bias in individual studies | 12 | Risk assessment was carried out using the Cochrane collaboration risk bias assessment tool. The evaluation included 1) Random sequence generation, 2) Allocation concealment, 3) Blinding of personnel, 4) Blinding of outcome assessment, 5) Incomplete outcome data, 6) Selective reporting, and 7) Other bias. The evaluation criteria are as follows: the “√” judgment is a low risk of bias, the “×” judgment is a high risk of bias, and the “?” judgment is an unclear risk of bias. Each study was based on an overall assessment of seven items, with a rating of high, moderate, and low risk. Two authors (T.H. and Z.H.) and trained research assistant separately estimate and cross-audit all articles using unified standards. Disgreements were resolved through discusion until there was 100% agreement. Statistical charts of risk bias were generated by RevMan 5.3 software. | 6 |
| Summary measures | 13 | In this review, a random-effect model was used for meta-analysis of the included studies, and STATA was used for analysis.Statistical analysis of data from different units was performed using a 95% confidence interval (95%CI) standardized mean difference (SMD). The weight means difference (WMD) of 95%CI was used for statistical analysis of data of the same unit. P < 0.05 was regarded as a significant difference. | 6-7 |
| Synthesis of results | 14 | The main analysis processes included forest map analysis, heterogeneity test, and subgroup analysis. | 6-7 |

Page 1 of 2

| **Section/topic** | **#** | **Checklist item** | **Reported on page #** |
| --- | --- | --- | --- |
| Risk of bias across studies | 15 | I^2^ statistics were used to test the heterogeneity between the included studies. When I^2^ < 25%, 25% - < 50%, 50% - < 75% and ≥ 75%, it was defined as none, low, moderate, and high degree of heterogeneity, respectively. Egger's test was utilized to detect whether there was a publication bias in the included study. Sensitivity analysis was conducted to test the robustness of the results, by replacing the fixed-effects model with the random-effects model and removing one study at a time to test whether a single study significantly modified the pooled effect. | 6-7 |
| Additional analyses | 16 | Depending on the characteristics of the included study, the subgroup analysis was conducted by outcomes, age, weight, means of intervention, contents of intervention , intervention duration, measuring methods, and study quality to test whether there were differences in the effects among different subgroups. | 6-7 |
| **RESULTS** | | |  |
| Study selection | 17 | A total of 1596 articles were searched from each database, 1585 articles were excluded according to the inclusion and exclusion criteria. Finally, 11 articles were included in this study.  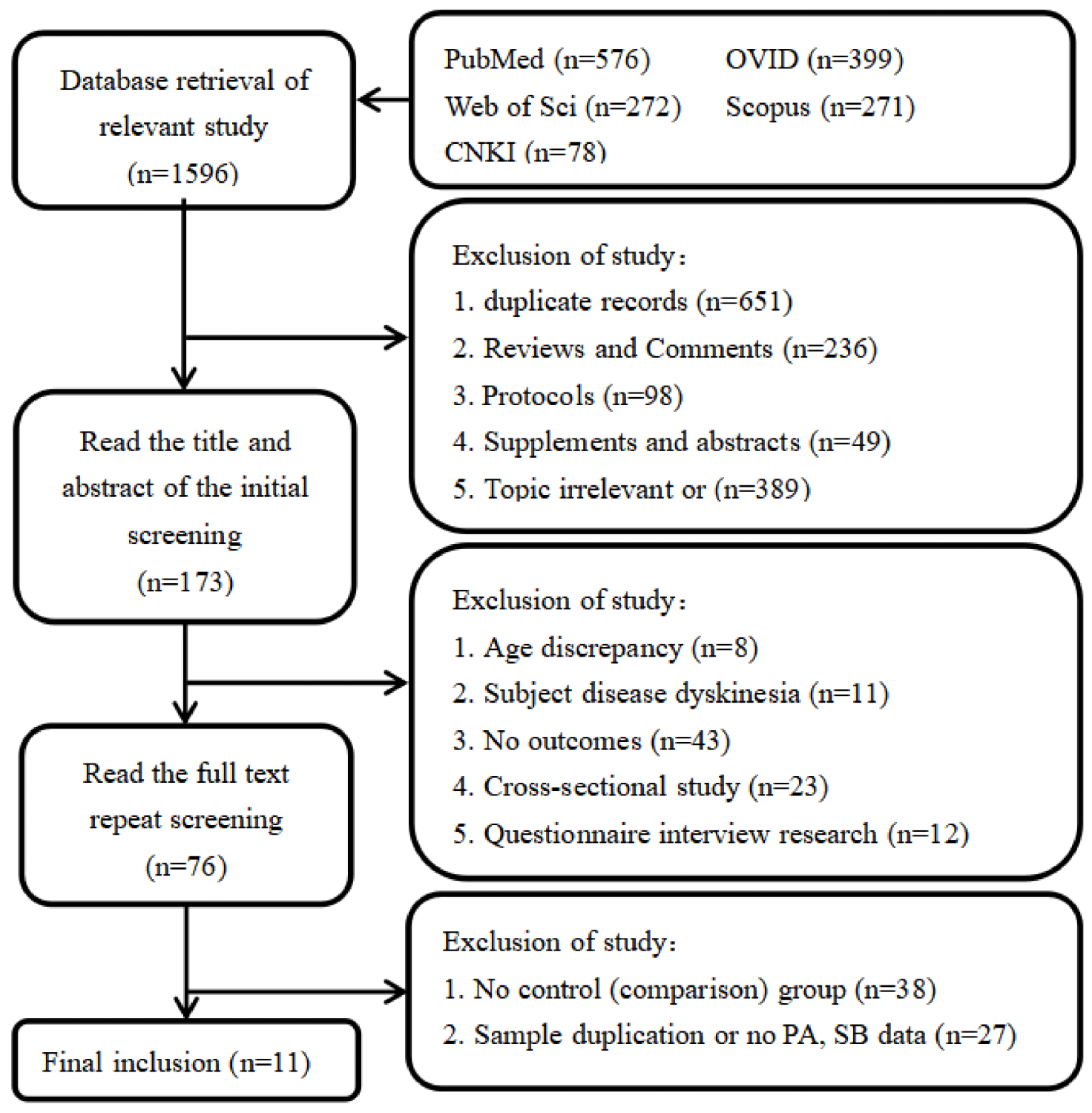 | 7 |
| Study characteristics | 18 | All of included studies were published in 2012 or later, among which 7 were published in 2015 or later. Of them, 4 studies from Australia, 3 from the United States. The United Kingdom, Germany, Finland, Norway and Sweden each have one study. The included studies consisted of 10 RCTs and 1 CCT, with a total of 955 participants in the treatment group and 931 participants in the control group. Five of the included studies only used theory interventions, including PA knowledge education, health behavior lectures, PA counseling services, interviews, and telephone return visits. One of the included studies38 only used behavior interventions in the form of specific activity tasks or activity classes that parents and children participated in together. In addition, 5 of the included studies used both theory and behavior interventions. Most interventions included in this review targeted more than one health behavior, and intervention focus were categorized as “PA only” and “included other behavior”. Intervention focus, “PA only”, focuses only on PA improvement during the intervention, not other health behaviors. “Included other behavior” focuses not only on PA but also on the improvement of other health behaviors (e.g. diet, sleep, screen time). | 7-8 |
| Risk of bias within studies | 19 | Of the 11 articles, 6 articles were classified as low risk, 2 articles were classified as moderate risk, and 3 articles were classified as high risk.  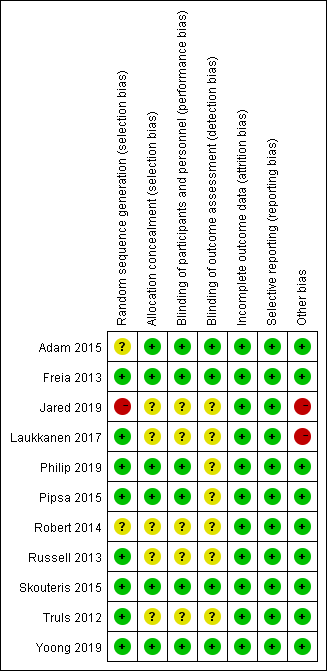 | 8-9 |
| Results of individual studies | 20 | 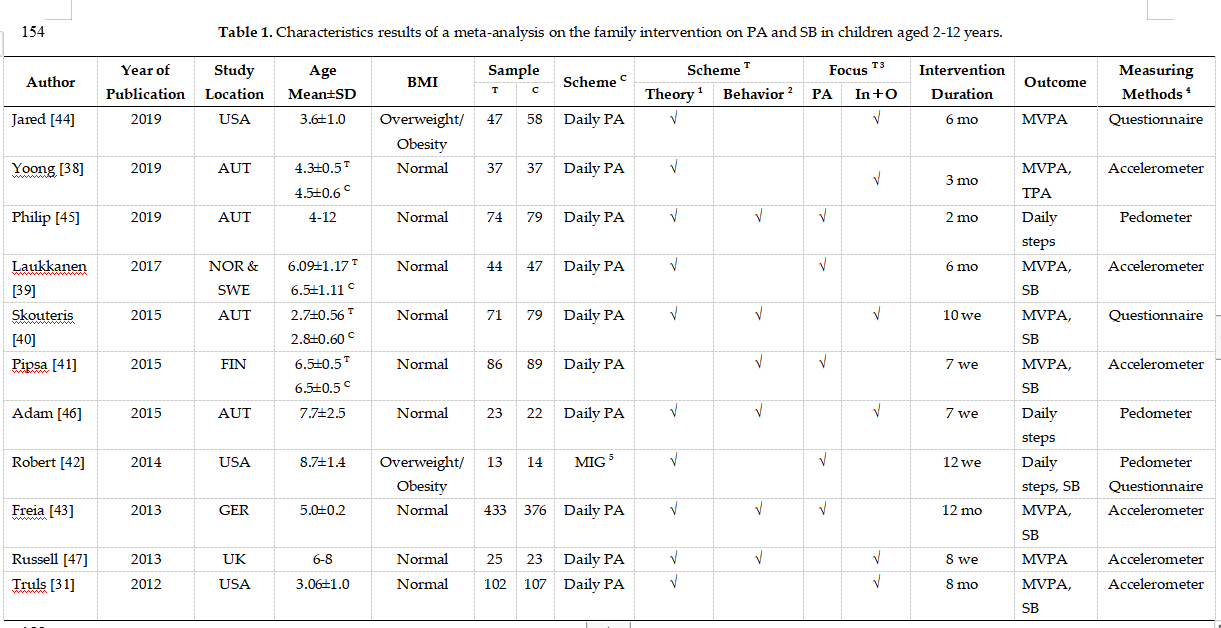 | 25 |
| Synthesis of results | 21 | Meta-analysis of 11 included studies was revealed that family intervention had a significant effect on the improvement of PA in children aged 2-12 years (SMD=0.10; 95%CI=0.01-0.19). Also, no significant heterogeneity was observed across included studies (I^2^=0%, P=0.52). Publication bias was also not observed with Egger's test (P=0.11).  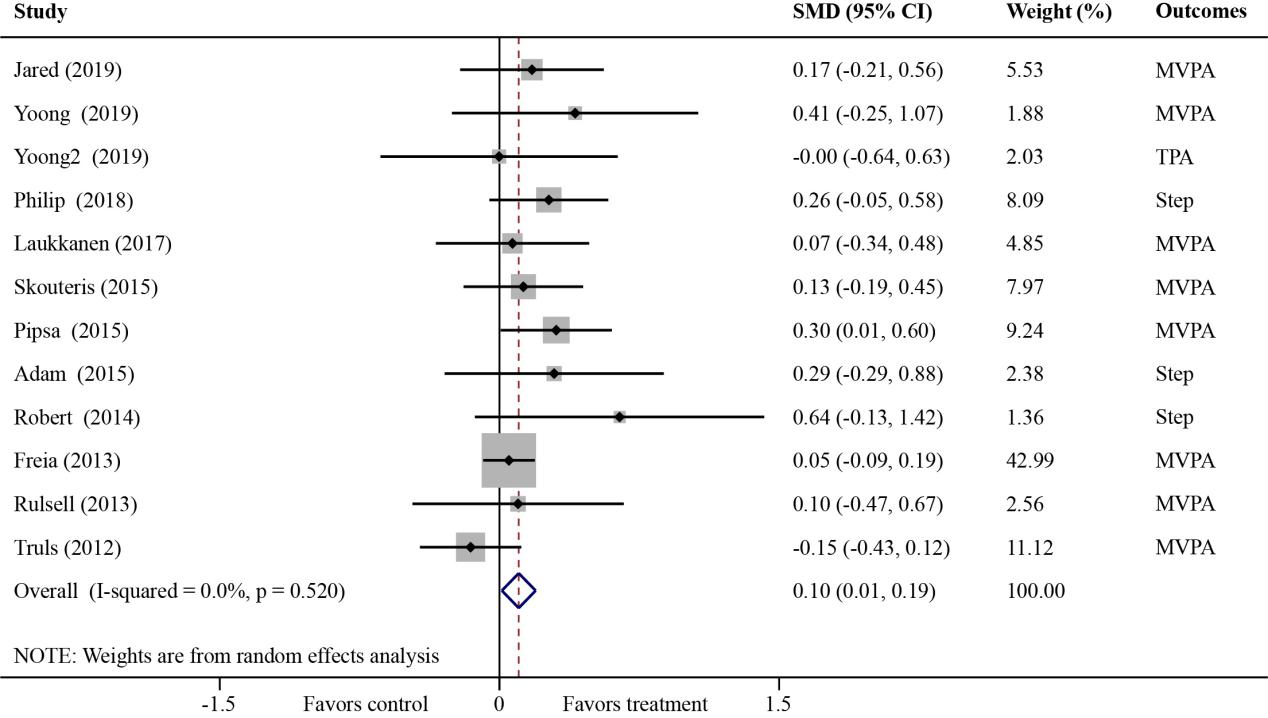  Subgroup analysis results showed that subgroups of “daily steps” (WMD=1,006; 95%CI=209-1,803), the “≥ 6 years” (SMD=0.24; 95%CI=0.04-0.45), intervention focus “PA only” (SMD=0.16; 95%CI=0.01-0.30), intervention duration “≤ 10 weeks” (SMD=0.25; 95%CI=0.09-0.41), and “low risk of bias” (SMD=0.13; 95%CI=0.02-0.23) have significant effect in PA promotion.  **Table 2.** Subgroup analysis of the effect of the family intervention on PA   | **Subgroup** | **Potential modifiers** | **No. of studies** | **Effect size (95%CI)** | **Heterogeneity** | | --- | --- | --- | --- | --- | | All studies |  | 11 | **0.10 (0.01-0.19)** | *I²*=0%, *P*=0.52 | | Outcomes ^1^ | MVPA | 8 | 0.43 (-1.19-2.04) | *I²*=19.6%, *P*=0.27 | | TPA | 1 | ———— | —— | | Daily steps | 3 | **1006** **(209-1803)** | *I²*=0%, *P*=0.86 | | Age ^2^ | < 6 years | 5 | 0.05 (-0.06-0.15) | *I²*=0%, *P*=0.56 | | ≥ 6 years | 5 | **0.24 (0.04-0.46)** | *I²*=0%, *P*=0.71 | | BMI | Normal | 9 | 0.09 (-0.003-0.19) | *I²*=0%, *P*=0.53 | | Overweight/Obesity | 2 | 0.28 (-0.11-0.67) | *I²*=11.6%, *P*=0.29 | | Types of intervention | Theory | 5 | 0.02 (-0.13-0.27) | *I²*=16.4%, *P*=0.31 | | Behavior | 1 | ———— | —— | | Theory puls behavior | 5 | 0.10 (-0.02-0.21) | *I²*=0%, *P*=0.74 | | Intervention focus | PA only | 5 | **0.16 (0.01-0.30)** | *I²*=20.6%, *P*=0.28 | | PA puls others | 6 | 0.06 (-0.10-0.22) | *I²*=0%, *P*=0.59 | | Intervention duration | > 10 we | 7 | 0.08 (-0.04-0.19) | *I²*=7.5%, *P*=0.37 | | ≤ 10 we | 4 | **0.22 (0.02-0.41)** | *I²*=0%, *P*=0.84 | | Measuring methods | Subjective | 2 | 0.15 (-0.10-0.39) | *I²*=0%, *P*=0.86 | | Objective | 9 | 0.11 (-0.004-0.22) | *I²*=9.5%, *P*=0.36 | | Risk of bias | Low risk | 6 | **0.13 (0.02-0.23)** | *I²*=0%, *P*=0.62 | | Moderate risk | 2 | -0.11 (-0.35-0.14) | *I²*=0%, *P*=0.43 | | High risk | 3 | 0.19 (-0.08-0.45) | *I²*=0%, *P*=0.44 |   *NOTE:* 1 The subgroup of outcomes units were the same, and WMD statistics were used, SMD was used for all the other subgroup except the outcomes subgroup. Yoong [38] contained two outcomes (MVPA and LPA), so the total number of outcomes subgroups was 12; 2 philip [40] is not divided into age subgroup because of participants were 4-12 years old.  Meta-analysis of 6 included studies was revealed that family intervention had no significant effect on the improvement of SB outcome in children aged 2-12 years (WMD=-0.38; 95%CI=-7.21-6.46). There was no significant difference in all subgroups. Also noteworthy was the fact that no significant heterogeneity was observed (I^2^=0%, P=0.82). Publication bias was also not observed with Egger's test (P=0.72). 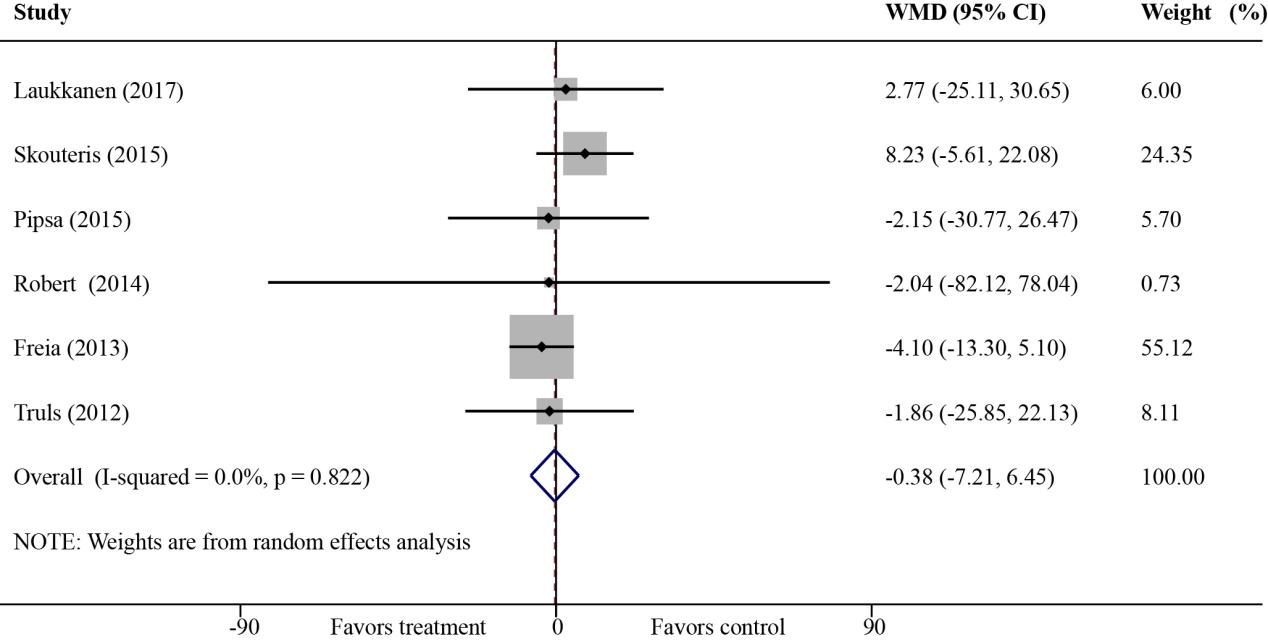  **Table 3.** Subgroup analysis of the effect of the family intervention on SB   | **Subgroup** | **Potential modifiers** | **No. of studies** | **WMD (min/day) (95%CI)** | **Heterogeneity** | | --- | --- | --- | --- | --- | | All studies |  | 6 | -0.38 (-7.21-6.46) | *I²*=0%, *P*=0.82 | | Age | < 6 years | 3 | -0.46 (-7.76-6.84) | *I²*=6%, *P*=0.35 | | ≥ 6 years | 3 | 0.23 (-19.15-19.61) | *I²*=0%, *P*=0.97 | | BMI | Normal | 5 | -0.37 (-7.22-6.49) | *I²*=0%, *P*=0.70 | | Overweight/Obesity | 1 | ———— | —— | | Types of intervention | Theory | 3 | -0.004 (-17.73-17.74) | *I²*=0%, *P*=0.97 | | Behavior | 1 | ———— | —— | | Theory plus behavior | 2 | -0.32 (-7.99-7.34) | *I²*=52.7%, *P*=0.15 | | Intervention focus | PA only | 4 | -3.30 (-11.62-5.01) | *I²*=0%, *P*=0.98 | | PA plus others | 2 | 5.71 (-6.28-17.70) | *I²*=0%, *P*=0.48 | | Intervention duration | > 10 weeks | 4 | -3.23 (-11.40-4.94) | *I²*=0%, *P*=0.97 | | ≤ 10 weeks | 2 | 6.27 (-6.20-18.73) | *I²*=0%, *P*=0.52 | | Measuring methods | Subjective | 2 | 7.94 (-5.71-21.58) | *I²*=0%, *P*=0.80 | | Objective | 4 | -3.16 (-11.05-4.73) | *I²*=0%, *P*=0.97 | | Risk of bias | Low risk | 3 | -0.44 (-7.85-6.96) | *I²*=6.1%, *P*=0.35 | | Moderate risk | 1 | ———— | —— | | High risk | 2 | 2.25 (-24.08-28.58) | *I²*=0%, *P*=0.91 | | 9 |
| Risk of bias across studies | 22 | All included studies were non-selective and the integrity of the data results was described in detail. More than half of all articles described randomization, allocation concealment, and blind implementation.  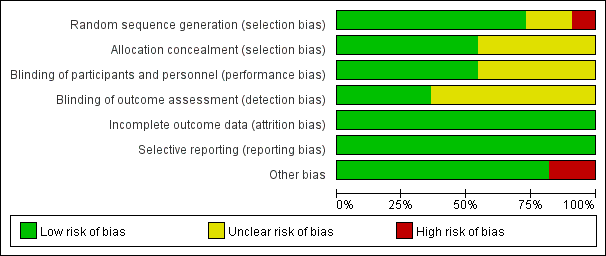 | 8-9 |
| Additional analysis | 23 | To test the robustness of the results, the fixed-effect model was used to replace the random-effect model to conduct statistics on all the consequences (including the combined effect and all the subgroup analysis), showing that the statistical results obtained by the two models were consistent. Further sensitivity analysis was conducted to test the robustness of the results pertaining to the combined effect by removing one study at a time to test whether a single study significantly modified the pooled effect, the results shown that the removal of any study had no significant effect on the combined effect. | 10 |
| **DISCUSSION** | | |  |
| Summary of evidence | 24 | This study aimed to quantitatively examine the effect of family interventions on the PA and SB in children aged 2-12 years by synthesizing the available literature in this field of inquiry. Through combined 11 included studies, we found that family intervention could effectively improve the PA of children aged 2-12 years, especially for daily steps, but there was no obvious effect on SB. | 10 |
| Limitations | 25 | This study has demonstrated several strengths. First, to the best of our knowledge, this is the first meta-analysis to quantitatively examine the effect of family interventions on PA in children aged 2-12 years, which provides additional insight in the field of family interventions and PA. Second, the meta-analysis is based on data from controlled trials studies regarded as a study design that substantially reduces selection bias and has a good comparability.  There were also some limitations in this study. First, most of the included studies was distributed in developed countries, so the research results were not widely representative. However, this study has included as much as possible the latest and most comprehensive research related to this proposition. Second, the family intervention programs (focus, means, duration) varied across included studies, which may lead to estimation bias of the overall effect. However, sensitivity analysis showed that the reduction of any one of the included studies did not significantly affect the combined results of this study. | 13-14 |
| Conclusions | 26 | In summary, findings from this meta-analysis that family intervention can effectively improve PA of children aged 2-12 years, especially daily steps, but has no obvious effect on SB. Considering that family members engage in physical activity together is safe, meaningful, and effective for not only promoting the relationship between parents and children but also development of good habits, we should encourage family members to take up physical exercise together. Future studies should focus on considering the different characteristics of preschoolers and school-age children, exploring the optimal combination of interventions focus, means, and duration. | 14 |
| **FUNDING** | | |  |
| Funding | 27 | This work was supported by National Natural Science Foundation of China (81703252, to M.Q.), the Shanghai Commission of Science and Technology (19080503000, to M.Q.), the “the Ministry of Education Research of Social Sciences Youth funded projects” of China (18YJC890060, to G.Z.), and “2019 Nanchang University Graduate student Innovation Fund” (CX2019046, to T.H.). | 15 |

*From:*  Moher D, Liberati A, Tetzlaff J, Altman DG, The PRISMA Group (2009). Preferred Reporting Items for Systematic Reviews and Meta-Analyses: The PRISMA Statement. PLoS Med 6(6): e1000097. doi:10.1371/journal.pmed1000097

For more information, visit: **www.prisma-statement.org**.

Page 2 of 2
